# Jejunojejunal intussusception after Roux-en-Y gastric bypass in a situs inversus totalis patient: A case report

**DOI:** 10.1097/MD.0000000000006589

**Published:** 2017-04-14

**Authors:** Guangyi Jiang, Baojuan Fu, Sheng Lv, Junjie Hong, Xiujun Cai

**Affiliations:** a Department of General Surgery; b Key Laboratory of Laparoscopic Technology of Zhejiang Province, Sir Run Run Shaw Hospital, College of Medicine, Zhejiang University, Hangzhou; c Department of General Surgery, Guang Fu Hospital, Jinhua, Zhejiang, China.

**Keywords:** abdominal pain, complication, intussusception, Roux-en-Y gastric bypass, situs inversus totalis

## Abstract

**Introduction:**

Situs inversus totalis (SIT) is an uncommon clinical manifestation. Patients with SIT typically have malformation in the thorax and abdomen. The incidence of SIT ranges from 1/10,000 to 1/20,000 (Al-Jumaily and Hoche. J Laparoendosc Adv Surg Tech A 2001;11:229). Jejunojejunal intussusception is a rare complication after Roux-en-Y gastric bypass. Intussusception in adult cases accounts for 5% of adult intestinal obstruction cases, while in children, the occurrence is high and the majority of them are idiopathic cases.

**Case report:**

Here, we present an uncommon case of jejunojejunal intussusception after Roux-en-Y gastric bypass in an SIT patient. We performed reduction at the beginning and resection was done finally.

**Discussion:**

We explore the potential causes and discuss the diagnosis and therapy.

**Conclusion:**

Intussusception in an SIT patient is an uncommon case. The symptoms are vague, and it is difficult to diagnose. Clinicians should be vigilant postoperatively, especially when abdominal pain after gastrointestinal surgery occurs. It is a rare case worth learning.

## 1 Introduction

Situs inversus totalis (SIT) is a rare congenital visceral malformation with total organ antiposition in abdominal and thorax, which we call mirror image. SIT is acknowledged as autosomal recessive but also related with X-link.^[1]^ The incidence of SIT ranges from 1/10,000 to 1/20,000.^[2]^ Patients with SIT usually appear normal, although some are at risk for Kartagener triad (bronchiectasis, sinusitis, and situs inversus).

Roux-en-Y gastric bypass is widely performed in gastrointestinal tumor and bariatric surgery. The reoperation rate is 3% to 20% due to long-term postoperative complication.^[3]^ Intussusception is an uncommon complication after Roux-en-Y gastric bypass. In children, intussusception is a major cause of bowel obstruction, while most of them are idiopathic. Intussusception in adult cases account for 5% of adult intestinal obstruction.^[4]^ Intussusception after gastric bypass typically has no lead point. According to a published report, most intussusceptions after Roux-en-Y gastric bypass are retrograde.^[3]^

In this paper, we provide a case of jejunojejunal intussusception after Roux-en-Y gastric bypass in an SIT patient. To the best of our knowledge, this is the first case of jejunojejunal intussusception after Roux-en-Y gastric bypass in an SIT patient in the literature.

## 2 Case report

**Figure 1:**
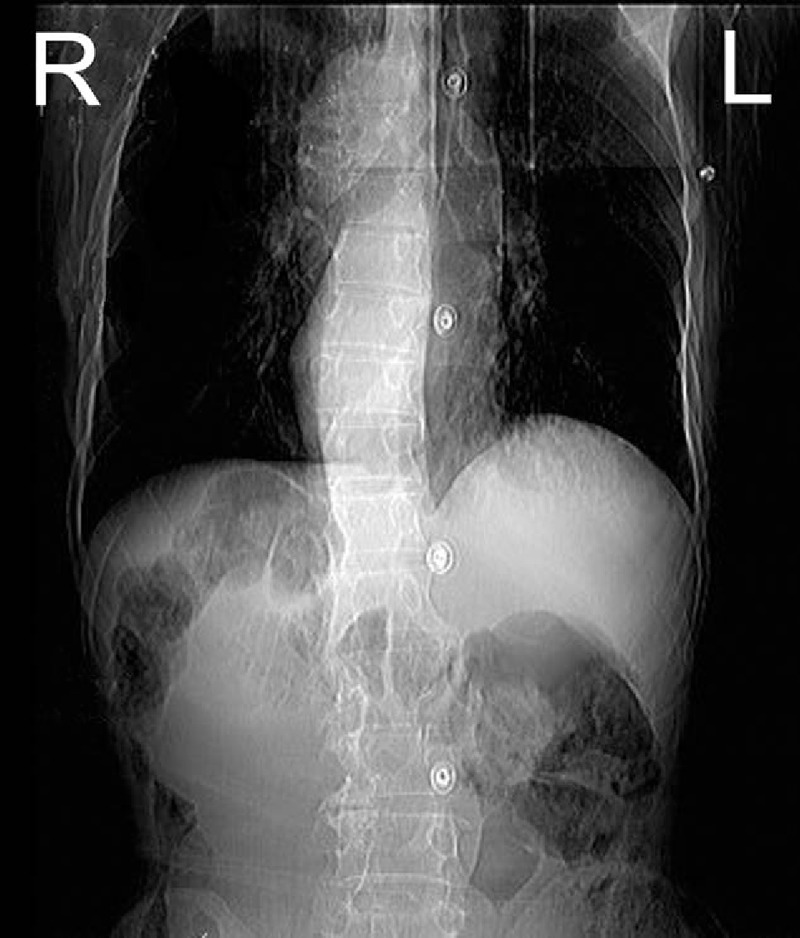
Plain film showed a left–right transposition of all viscera and dilated intestine.

**Figure 2:**
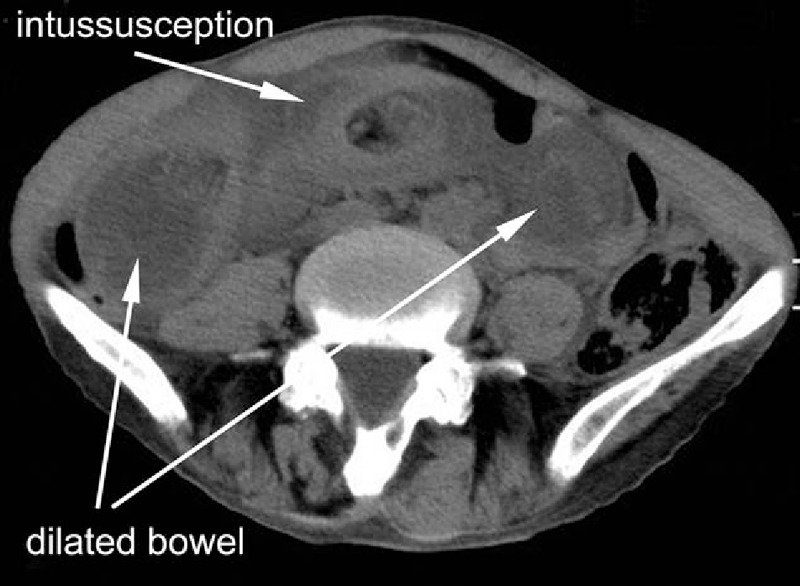
Computed tomography showed a typical image of intussusception, together with dilated jejunal loops.

**Figure 3:**
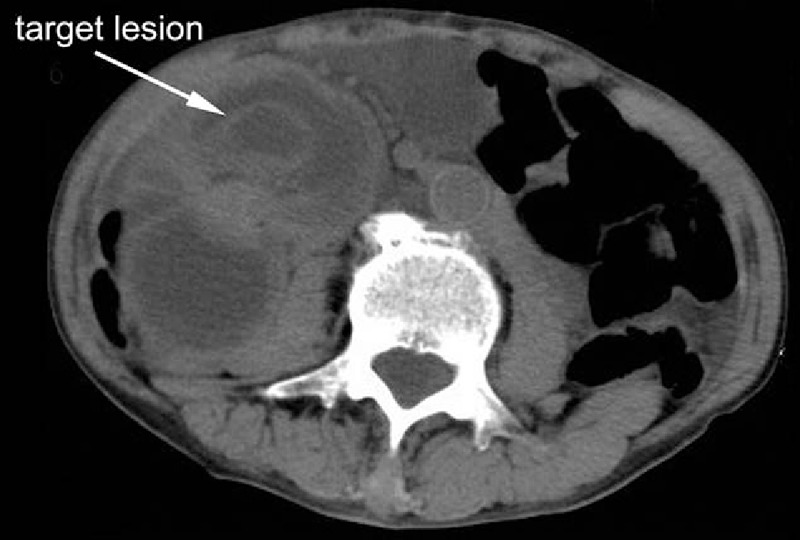
A target lesion was showed, suggestive of obstruction.

A 69-year-old male came to the emergency department with a 1-week history of abdominal distension and 1-day history of nausea and vomiting. Small amount of stomach content had been vomited during several episodes of hematemesis. Abdominal pain was paroxysmal, and the pain exceeded a normal pain tolerance. Defecation had stopped since vomiting. Five years ago, the patient was admitted for total gastrectomy due to stomach adenocarcinoma at gastric angle, and Roux-en-Y anastomosis was performed for digestive tract reconstruction. After surgery, the patient accepted chemotherapy. He had a known history of situs inversus. Plain X-ray showed a left–right transposition of all viscera as well as dilated intestine (Fig. 1). Physical examination showed a palpable, intestine-like mass and abdominal tenderness. Obstruction was considered due to the history. Computed tomography (CT) showed a target lesion and dilated bowel (Figs. 2 and 3) suggestive of intussusception.

**Figure 4:**
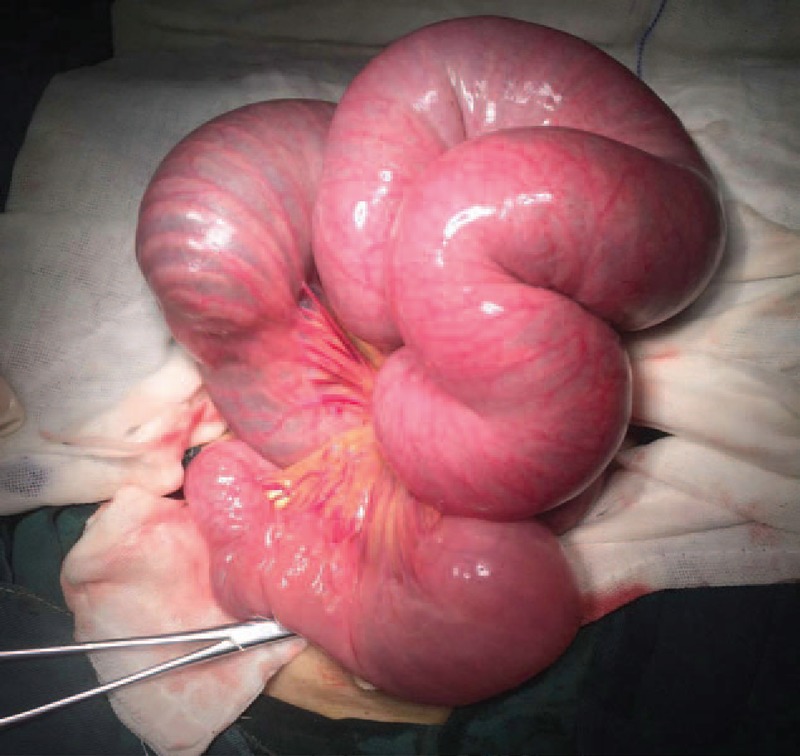
Surgical finding showed dilated and edematous jejunum.

**Figure 5:**
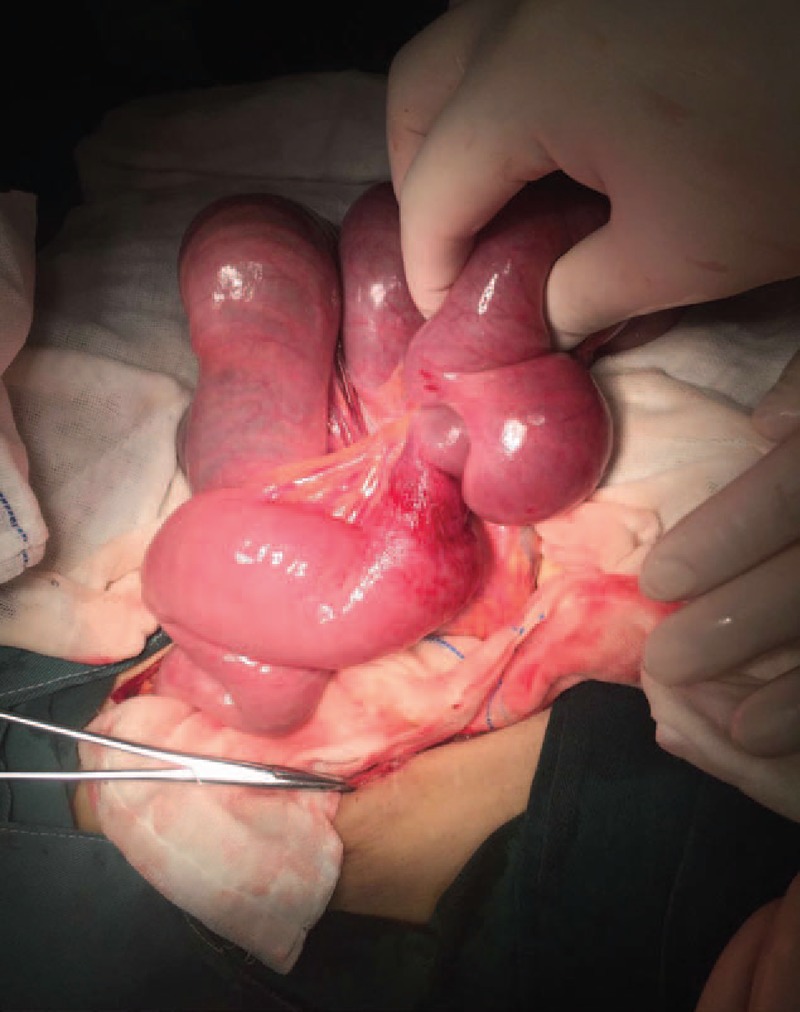
Partial bowel was reduced, and ecchymosis was showed.

**Figure 6:**
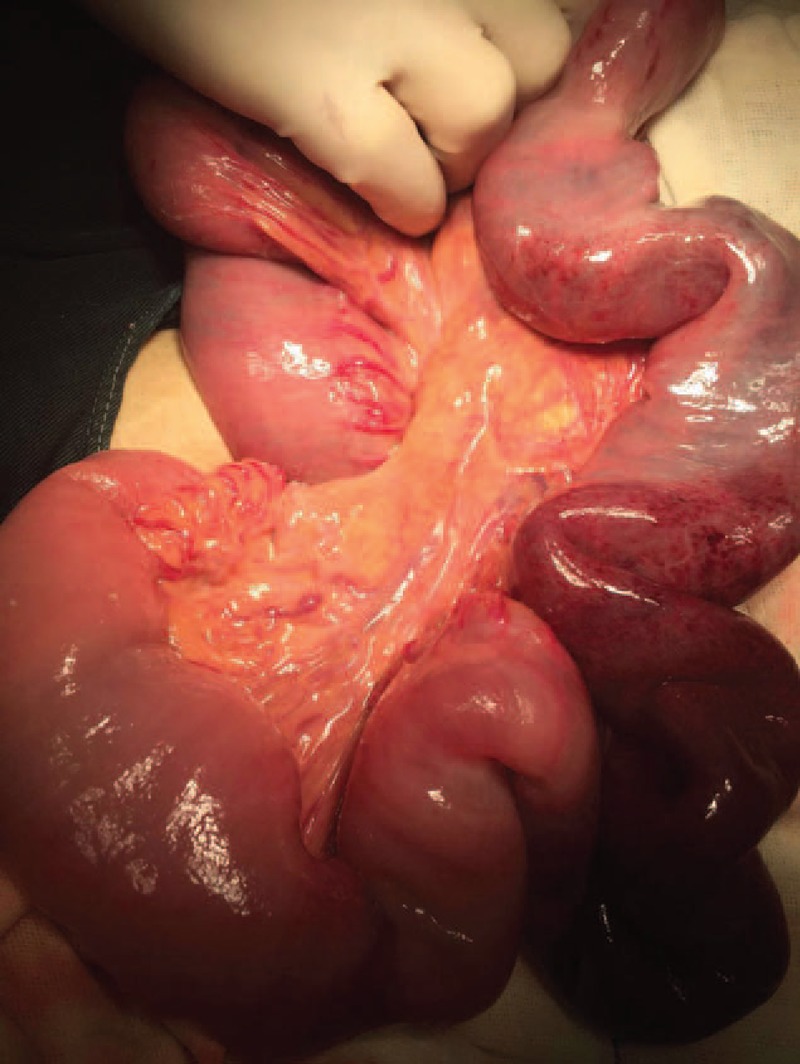
As is shown above, we performed reduction. The perfusion of invaginated segment is insufficient.

Therefore, an emergency operation was performed. Surgical finding showed a jejunojejunal retrograde intussusception. The distal jejunum, which was 30 cm away from the anastomosis telescope into the proximal jejunum, exceeded the anastomosis. The length of the invaginated segment was 35 cm. The bowel was significantly dilated and edematous with exudation (Fig. 4). We performed reduction at the beginning because the bowel was not in complete necrosis (Fig. 5). We waited to observe if the bowel could perform reperfusion (Fig. 6). According to our experience, recovery of the bowel perfusion was challenging; hence, resection of necrotic jejunum and end-to-end anastomosis was performed. The postoperative course was uneventful. After discharged, he remained quite well.

## 3 Discussion

SIT is a rare clinical entity, and the coincidence of jejunojejunal intussusception after Roux-en-Y gastric bypass, along with SIT, has not been reported. SIT patients are typically as healthy as normal people and asymptomatic. However, malformation is not uncommon in SIT patients, as according to available literature, SIT patients presenting with Kartagener triad account for 20% to 25% of clinical SIT.^[5]^

The lead point that causes intussusception is rarely in the postoperative cases, although abnormal alimentary canal occurs at times and may cause abnormal peristalsis, which can contribute to intussusception. Hocking et al^[6]^ studied bowel peristalsis and first suggested that intussusception might be associated with intestine motility disturbances. Their study indicated 2 types of propagation (orad-propagated and aboard-propagated) regulation disturbances that may cause intussusception. Reverse intussusception, also called retrograde intussusception, is a rare incident that moves the peristalsis process distally to proximally.^[7]^ Daellenbach and Suter^[3]^ collected 63 cases of postoperative intussusception after Roux-en-Y gastric bypass and suggested that high pressure of the roux limb and 2 opposite direction waves, which produced by normal pacesetter and potential pacesetter, act as lead points. In our case, the patient presented with abdominal distension for 1 week, and surgical finding showed a large dilated jejunum, and though the exact etiology is still unknown, we speculate that high pressure of enteric cavity may be associated with the abnormal motility that existed for a period of time.

Goverman et al^[8]^ studied retrograde intussusception and hypothesized that “Roux stasis syndrome” may be associated with retrograde intussusception. The cause of roux stasis syndrome may correlate with the disorder of electrical conduction caused by cutting of the gut. The potential pacemaker may activate due to losing the normal pacemaker, which may create a duo-directional current.^[9,10]^ The reverse phase current caused the symptoms of abdominal pain, such as nausea or vomiting. Therefore, we assumed that the Roux stasis syndrome is a precursor of intussusception. Intussusception, associated with spontaneous reduction, often has slight symptoms and even no clinical presentation. Sometimes, patients just present with abdominal distention and recover soon, rather than recurrent intussusception patients, which present with intensive pain that is hard to reduce.

Diagnosis is difficult by physical examination, especially in chronic cases. CT is a sensitive examination used to diagnose intussusception. With the development of the resolution ratio of CT, more transient intussusceptions had been diagnosed.^[11]^ High-resolution-ratio CT also has identified pathological causes, and it may serve as the leading point.^[4]^ Gayer et al^[12]^ suggested in his article that CT is one of the most reliable examinations for diagnosing, with the characteristic target lesion “double ring.” Ultrasound is useful when patients present with a palpable mass in the abdomen, as convenience and high accuracy rating is the advantage. Urgent treatment is vital. In cases such as ours, surgical procedure is recommended, especially when the clinical signs suggest bowel necrosis, in light of the high recurrence rate of reduction. Radiographic images must be examined carefully before the operation as SIT patients may have an anatomic abnormality. Blind operation can lead to severe consequences. Some cases of intussusception recover spontaneously or just have slight clinical symptoms. Various methods of reduction are available, and in our case, handy reduction had been performed, and owing to the risk of bowel necrosis and recurrence, we performed resection and end-to-end anastomosis.

In clinical practice, we typically meet the situation that patients postoperatively present with abdominal pain. Identifying the etiology is an important step to conduct therapy. Major causes of abdominal pain are peritoneal adhesion. According to available literature, the possibility of patients who underwent low abdominal surgery developing adhesion is 67% to 93%, but very few become symptomatic.^[13]^ The interval between initial operation and presented symptom (obstruction) is 8 days to 60 years, with an average of 3.7 to 8.9 years.^[14]^ Intussusception caused by adhesion is rare but must be taken into consideration when patients present with postoperative abdominal pain. Thus far, there is no technique, either chemotherapy or surgical method, that is efficient at reducing the risk of postoperative adhesion.

## 4 Conclusion

SIT is a congenital visceral malformation and has an extremely low occurrence. Intussusception in an SIT patient is an uncommon case. The symptoms are vague, and it is difficult to diagnose by physical examination alone, and the exact etiology is still unknown. We suppose that the abnormal motility of bowel and postoperative adhesion leads to intussusception. Correlation between the visceral inversion and intussusception needs further investigation. CT is sensitive for diagnosis, and operation is the recommended therapy in severe cases.
